# Continuous intraoperative monitoring of pelvic autonomic nerves during TME to prevent urogenital and anorectal dysfunction in rectal cancer patients (NEUROS): a randomized controlled trial

**DOI:** 10.1186/s12885-016-2348-4

**Published:** 2016-05-21

**Authors:** D. W. Kauff, K Kronfeld, S Gorbulev, D Wachtlin, H Lang, W Kneist

**Affiliations:** Department of General, Visceral and Transplant Surgery, University Medicine of the Johannes Gutenberg-University, Mainz, Germany; Interdisciplinary Center for Clinical Trials (IZKS), University Medicine of the Johannes Gutenberg-University, Mainz, Germany; Boehringer Ingelheim Pharma GmbH & Co. KG, Ingelheim, Germany

**Keywords:** Rectal cancer, Autonomic nerves, Intraoperative monitoring, Urinary dysfunction, Sexual dysfunction, Fecal incontinence, Quality of life

## Abstract

**Background:**

Urinary, sexual and anorectal sequelae are frequent after rectal cancer surgery and were found to be related to intraoperative neurogenic impairment. Neuromonitoring methods have been developed to identify and preserve the complex pelvic autonomic nervous system in order to maintain patients’ quality of life. So far no randomized study has been published dealing with the role of neuromonitoring in rectal cancer surgery.

**Methods/design:**

NEUROS is a prospective two-arm randomized controlled multicenter clinical trial comparing the functional outcome in rectal cancer patients undergoing total mesorectal excision (TME) with and without pelvic intraoperative neuromonitoring (pIONM). A total of 188 patients will be included. Primary endpoint is the urinary function measured by the International Prostate Symptom Score. Secondary endpoints consist of sexual, anorectal functional outcome and safety, especially oncologic safety and quality of TME. Sexual function is assessed in females with the Female Sexual Function Index and in males with the International Index of Erectile Function. For evaluation of anorectal function the Wexner-Vaizey score is used. Functional evaluation is scheduled before radiochemotherapy (if applicable), preoperatively (baseline), before hospital discharge, 3 and 6 months after stoma closure and 12 months after surgery. For assessment of safety adverse events, the rates of positive resection margins and quality of mesorectum are documented.

**Discussion:**

This study will provide high quality evidence on the efficacy of pIONM aiming for improvement of functional outcome in rectal cancer patients undergoing TME.

**Trial registration:**

Clinicaltrials.gov: NCT01585727. Registration date is 04/25/2012

## Background

Urinary, sexual and anorectal functional disturbances do frequently occur after total mesorectal excision (TME) for rectal cancer and may tremendously impair patients’ quality of life. In order to reduce the dysfunction rates it is recommended to identify and preserve the pelvic autonomic nerves during the surgical procedure. However, visual nerve identification especially of those located in the minor pelvis (inferior hypogastric plexus, pelvic splanchnic nerves and neurovascular bundles) is challenging due to the complexity of neural distribution and further patient as well as surgery related factors such as a narrow or deep pelvic cavity, the appearance of a voluminous mesorectum, severe obesity, previous pelvic surgery, neoadjuvant chemoradiotherapy, locally advanced tumors with anterior quadrant involvement, supra-anal or juxta-anal tumors, adherence or infiltration of adjacent organs, a bloody situs, use of additional diathermy coagulation and the applied dissection techniques [1–4]. Therefore, poor nerve visualization and lack of neuroanatomical knowledge will consequently result in inadvertent nerve damage.

Pelvic intraoperative neuromonitoring (pIONM) was introduced to rectal cancer surgery to serve as a novel method for improvement of nerve identification and further verification of its functional integrity. In previous investigations it could be already shown that the electrophysiological nerve identification is superior to sole visual assessment [5]. A recently developed pIONM method is based on electric stimulations of pelvic autonomic nerves under simultaneous observation of processed electromyography (EMG) of the internal anal sphincter (IAS) and manometry of the urinary bladder. Its suitability for accurate assessment of nerve function and its predictive potential of functional outcome has been demonstrated by previous non-randomized single-center studies [6, 7]. In a recent case-control study pIONM controlled TME in rectal cancer patients was found to offer better functional outcome compared to patients undergoing surgery without the use of this method [8]. An actual retrospective investigation supports these findings by demonstrating superior urogenital function in patients undergoing rectal cancer surgery with electrophysiological control [9]. The results are encouraging. However, high quality evidence on the efficacy of pIONM is missing. In order to close the gap, we are conducting the first randomized multicenter trial comparing urogenital and anorectal functional outcome in rectal cancer patients undergoing TME with or without pIONM.

## Methods/design

### Objectives

The primary objective of this trial is to assess urinary functional outcome in rectal cancer patients undergoing TME with pIONM, when compared to TME without pIONM, in a 12 months follow-up period per patient. The secondary objectives are to assess sexual and anorectal functional outcome in rectal cancer patients undergoing TME with pIONM, when compared to TME without pIONM, in a 12 months follow-up period per patient and to assess the safety, especially oncologic safety and quality of TME.

### Trial design

The NEUROS study is a prospective two-arm randomized controlled multicenter clinical trial with a follow-up period of 12 months per patient.

#### Centers currently participating:

Department of General Visceral and Transplant Surgery, University Medicine of the Johannes Gutenberg-University Mainz, Germany

Department of General and Visceral Surgery, University Medical Center Göttingen, Germany

Department of General Surgery and Centre for Minimally Invasive Surgery, Hannover Hospital (Siloah), Germany

Department of General Surgery, Schwarzwald-Baar-Klinikum, Teaching Hospital of the University of Freiburg, Villingen-Schwenningen, Germany

Department of General, Visceral, Transplantation, Vascular and Thoracic Surgery, Ludwig Maximilians University (LMU), Munich, Germany

Department of Surgery, University of Schleswig-Holstein (UKSH), Campus Lübeck, Germany

Department of Visceral, Transplant, Thoracic and Vascular Surgery, University Hospital of Leipzig, Germany

### Study population

Patients undergoing TME for rectal cancer presenting at one of the participating hospitals.

#### Inclusion criteria

- Histologically confirmed rectal cancer (≤ 16 cm from anal verge)
- Suitable for radical surgery
- TME
- Age 18-90 years
- Informed consent

#### Exclusion criteria

- Emergency operation
- Pacemaker
- Multivisceral resection
- Partial mesorectal excision
- Transanal endoscopic microsurgery
- Ongoing infection or sepsis
- Severe untreated physical or mental impairment
- Pregnancy or breastfeeding
- Women of childbearing potential who are not using a highly effective birth control method
- Missing preoperative data on urogenital or anorectal function
- Simultaneous participation in another clinical trial
- Previous participation in this clinical trial
- Lack of compliance with the trial procedure

### Outcome measures

Assessment of urinary function is carried out on the basis of the International Prostate Symptom Score (IPSS, total score range from 0 to 35 points) and the Quality of life index (Qol, Quality of life due to urinary symptoms, total score range from 0 to 6 points) [10]. A higher score indicates diminished urinary function and quality of life. Previous studies reported that the IPSS also applies to females and demonstrated that women have comparable scores to those of age-matched men [11, 12].

Sexual function in females is evaluated with the Female Sexual Function Index (FSFI) and in males with the International Index of Erectile Function (IIEF). The FSFI has been developed and validated as a brief, multidimensional self-report instrument for assessing the key dimensions of sexual function in women. It was designed and validated for assessment of female sexual function and quality of life in clinical trials and has demonstrated ability to discriminate between clinical and non-clinical populations [13]. The FSFI is a 19-item questionnaire with a total score range from 2 to 36 points. A lower score indicates diminished sexual function. The IIEF has been developed and validated as a brief and reliable self-administered scale for assessing erectile function [14]. The IIEF is a 19-item questionnaire with a total score range from 5 to 75 points. The brevity and ease of comprehension of the measure provide practical advantages.

Anorectal function is determined by the Wexner-Vaizey score (WVS) (minimum score = 0 = perfect continence; maximum score = 24 = totally incontinent) [15].

### Sample size calculation

The rate of patients with an IPSS increased by at least 5 points 12 months after surgery compared to the preoperative IPSS is assumed to be 10 % in patients undergoing TME with pIONM. The corresponding rate for patients undergoing TME without pIONM is expected to be 30 %. A total number of 164 study patients is needed to demonstrate a significant difference between the study arms with a power of 90 % using Fisher’s exact test (alpha = 0.05, two-sided). Dropout rates are expected to be 2 % perioperatively and 10 % during the follow-up period in both study arms resulting in an overall dropout rate of 12 %. 188 patients have to be allocated to the trial.

### Withdrawal of study participants

Study participants can leave the study for the following reasons:

- On the own request of the patient
- On the direction of the investigator, if a further participation at the trial may be disadvantageous for patient’s health
- If exclusion criteria become known
- Pregnancy
- Non-compliance

The investigator can decide to withdraw a participant from the study for the above mentioned reasons. This will be documented in the case report form (CRF) and the physician is required to notify the Coordinating Investigator. In all cases, the reason for withdrawal must be recorded in the CRF and in the patient’s medical record. All patients who discontinue because of adverse events or clinical laboratory abnormalities should be followed up until they recover or stabilize, and the subsequent outcome should be recorded.

### Replacement of study participants

Patients who underwent randomization and were withdrawn will not be replaced.

### Stopping rules for the whole trial

- New risks for subjects become known.
- Medical or ethical reasons affecting the continued performance of the trial.

### Endpoints

#### Primary endpoint

Primary endpoint is the increase of the IPSS by at least 5 points observed 12 months after surgery compared to the preoperative IPSS per patient. In case of postoperative urologic treatment for newly developed urinary dysfunction, primary endpoint is the increase of the IPSS by at least 5 points observed before urologic treatment compared to the preoperative IPSS. The primary endpoint is based on previous findings [16]. In a group of 61 patients undergoing mesorectal excision for rectal cancer we observed long-term urinary deterioration in 13 patients determined by the IPSS. The median difference in the IPSS was 6 points (range: 1-25 points, interquartile range: 4-8 points). Answers to additional questions on the Qol ranged from 0 (delighted) to 6 (terrible). We found that an increase of the IPSS by at least 5 points leads to a clear decrease in patients’ quality of life due to urinary symptoms (median difference in the Qol score was 3 points (interquartile range: 2-4 points)).

#### Secondary endpoints

Secondary endpoint is the reduction of FSFI/IIEF by at least 8/15 points 12 months after surgery compared to the preoperative FSFI/IIEF per patient. The secondary endpoint is analyzed separately for men and women. No confirmatory analyses are planned for this endpoint. Another secondary endpoint is the change of the WVS observed 12 months after surgery compared to the preoperative score per patient. For assessment of safety, especially oncologic safety adverse events, the rates of pCRM-positive specimen (distance of tumor from circumferential resection margin ≤ 1 mm) and the quality of TME will be documented. The quality of the mesorectum is scored using the M.E.R.C.U.R.Y. grading (Grade I: complete; Grade II: nearly complete; Grade III: incomplete) [17].

### Randomization and blinding

**Fig. 1:**
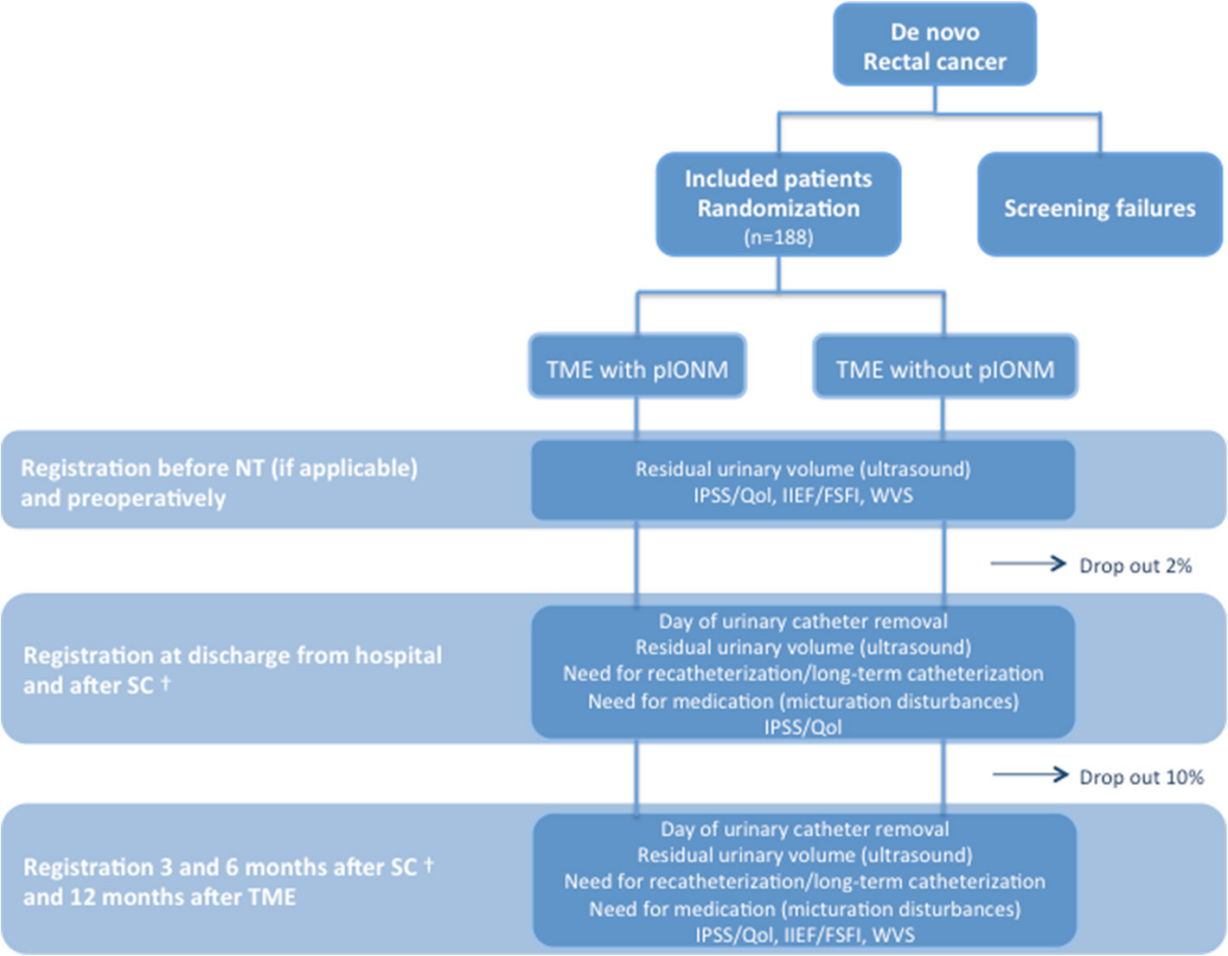
Summary of study interventions/flow diagram. † In patients who did not undergo stoma closure, study visits are planed 6 and 12 months after TME. Assessment of IIEF/FSFI and WVS will not be carried out. NT: Neoadjuvant therapy, TME: Total Mesorectal Excision, SC: Stoma closure, IPSS: International Prostate Symptom Score, Qol: Quality of life due to urinary symptoms, IIEF: International Index of Erectile Function, FSFI: Female Sexual Function Index, WVS: Wexner-Vaizey Score, pIONM: pelvic intraoperative neuromonitoring

All patients who meet the inclusion criteria will be randomized. Randomization ratio is 1:1 to TME with pIONM or TME without pIONM (Fig. 1). Randomization is stratified according to neoadjuvant therapy and gender using blocks of variable length. Central randomization via FAX is conducted in this study.

### Prescreening / Screening and follow up

**Fig. 2:**
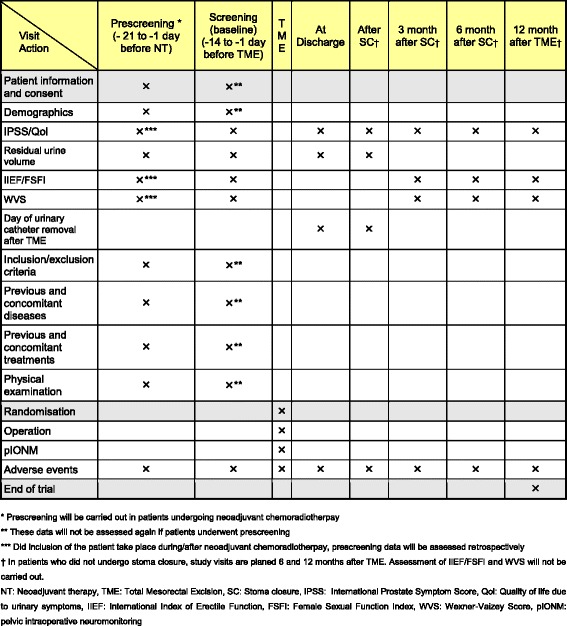
Frequency and scope of study visits

In patients undergoing neoadjuvant therapy a prescreening is scheduled 21 to 1 day before therapy begins. The preoperative screening (baseline) is scheduled 14 to 1 day before TME. Study visits and follow up are summarized in Fig. 2.

### TME and pelvic intraoperative neuromonitoring

#### Total mesorectal excision

In surgical treatment of rectal cancer, adequate mesorectal excision leads to an optimization of oncologic results. For cancer in the middle and lower rectal third (≤12 cm from the anal verge) a TME is recommended.

#### Neuromonitoring setup

Monitoring of the pelvic autonomic nerves is carried out with a neuromonitoring system (504012 ISIS Touch and accessories, CE 0297, inomed, Emmendingen, Germany), which enables electric stimulation under simultaneous observation of processed EMG signals of the IAS and manometry of the urinary bladder. To observe IAS activity, bipolar needle electrodes (530228, CE 0297, inomed, Emmendingen, Germany) are inserted transanally under endosonographic guidance. The ground electrode (530627, CE 0297, inomed, Emmendingen, Germany) is placed on the left gluteal muscle and the electrodes are connected to the neuromonitoring device. The impedance is measured to ensure correct placement. Simultaneous observation of intravesical pressure is carried out through the transurethral bladder catheter or if applicable suprapubic catheter. The catheter is connected to a pressure transducer (520320, CE 0297, inomed, Emmendingen, Germany), which is linked to the amplifier of the neuromonitoring device. Thereby both measurements could be continuously visualized as neuromonitoring signals online on the screen of the system. Before the onset of neurostimulation the bladder is emptied and filled with 200 ml of Ringers’ solution. Stimulation parameters are set to currents of 1-25 mA, frequency of 30 Hz, and monophasic rectangular pulses with pulse duration of 200 μs.

According to previous investigations a stimulation dependent unilateral or bilateral consecutive increase in intravesical pressure (cmH_2_O) or processed EMG amplitude (V) of IAS will be rated as positive response verifying functional integrity of urinary and anorectal innervation. With regard to sexual function bilaterally observed positive results by manometry and IAS-EMG were valued as preserved genital innervation [5, 6].

#### Pelvic intraoperative neuromonitoring (pIONM)

Stimulation of the pelvic autonomic nerve during mesorectal dissection is performed by the surgeon with a handheld bipolar microfork probe (EW0266 and 522027, CE 0297, inomed, Emmendingen, Germany) and served for identification and verification of functional nerve integrity.

Initial neurostimulations are carried out bilaterally after posterior/posterolateral mesorectal dissection in order to detect the pelvic splanchnic nerves. Therefore, bilateral repetitive stimulations along the pelvic sidewall are carried out (stimulation period 3-10 seconds and resting period in between the stimulations of 3-10 seconds) as a kind of neuromapping under continuous observation of processed IAS-EMG. During ongoing lateral mesorectal dissection neuromapping is performed under simultaneous manometry of the urinary bladder and IAS-EMG for identification of further potentially surgical exposed nervous tissue (pelvic splanchnic nerves S2-4, inferior hypogastric plexus).

Anterolateral mesorectal dissection is performed with neuromapping under continuous processed IAS-EMG. For quality assurance of the nerve-sparing technique after TME (resection of specimen), the autonomic innervation is finally verified by bilateral neuromapping along the pelvic sidewall and just above the pelvic floor under simultaneous manometry of the urinary bladder and IAS-EMG. All observed neuromonitoring signals are manually documented.

### Statistical analysis

The primary analysis population for the efficacy parameters is the intention-to-treat (ITT) population. The ITT population includes all randomized patients for which a preoperative and postoperative measurement of IPSS is available. Patients will be analyzed in the treatment group to which they were randomized. In addition, analyses for the Per-Protocol population will be performed. Only patients with a minimum of compliance to the study protocol will be included into the Per-Protocol population. Relevant violations of the study protocol will be defined in the statistical analysis plan, which is finalized before the database is closed and unblinded. Differences between rates of patients with an IPSS increased by at least 5 points 12 months after surgery compared to the preoperative IPSS will be evaluated using the Wilcoxon signed rank test (two-sided, alpha = 0.05). Patients for which no postoperative IPSS is available will be excluded from the confirmatory analysis of the primary endpoint due to missing information about postoperative urinary function. Missing IPSS scores 12 months after surgery will be imputed according to the Last Observation Carried Forward (LOCF) method if a postoperative IPSS is available. Postoperative IPSS measured under the influence of additional therapies against urinary dysfunction must not be used. Therefore, the last observed IPSS before urologic treatment will be analyzed. As dropout rates and imputed missing values are expected to be equal in both study arms no selection bias is expected by the application of these procedures. The secondary outcome parameters will be analyzed only descriptively. Preoperative IPSS, IPSS 12 months after surgery and intra-individual changes of IPSS within 12 months after surgery will be analyzed by distributional parameters such as mean, median, range and standard deviation separately for each study arm. An analogous analysis will be performed for the IIEF in male patients, the FSFI in female patients and the WVS. For male patients the rates of patients with an IIEF reduced by at least 15 points 12 months after surgery compared to the preoperative IIEF score will be displayed separately for each study arm. For female patients these rates will be calculated for the FSFI analogously. The threshold for discretizing the change in FSFI within 12 months after surgery is set to 8 points.

For the safety population summaries and listings of safety data will be performed. Frequencies of subjects experiencing at least one adverse event will be displayed by body system and preferred term according to MedDRA terminology. Detailed information collected for each adverse event will include: A description of the event, duration, whether the adverse event was serious, intensity, relationship to trial treatment, action taken and clinical outcome. Summary tables will present the number of subjects observed with adverse events and corresponding percentages. Additional subcategories will be based on event intensity and relationship to trial treatment. A subject listing of all adverse events will be prepared.

For the assessment of oncologic safety the rates of pCRM-positive specimen and the quality of mesorectum will be displayed by means of absolute and relative frequencies separately for each study arm.

### Ethical considerations

#### Assessment of risks and benefits

So far, there were no reports about differences in risk potential for patients undergoing intraoperative electrophysiological confirmation of pelvic autonomic nerves, especially with regard to life threatening events. The individual participant will therefore not run any additional risk during participation in this trial. The potential benefit for the group of patients with additional pIONM is the avoidance of pelvic autonomic nerve damage with maintenance of quality of life, respectively. The intraoperative assessment of nerve-sparing and the predictability of postoperative urogenital and anorectal functional disturbances may offer secondary prevention with the potential for an improved prognosis.

#### Care and protection of research participants

Nerve-sparing TME is a standard treatment for patients with rectal cancer. There are no special adverse events expected. Surrogate parameters of oncologic outcome (rates of R1 and R2 resections, rates of pCRM-positive specimen, and rates of incomplete mesorectal excision) will be closely monitored by the Data Safety Monitoring Board (DMSB). All adverse events and serious adverse events will be recorded. The serious adverse events will be reported within 24 hours of the initial observation to the Interdisciplinary Center for Clinical Trials (IZKS) at the University Medicine of the Johannes Gutenberg-University Mainz, Germany.

### Availability of data and materials

The access to the confident patient information may be granted only to the governmental bodies and authorized representatives of the trial sponsor (clinical monitors). Only patients who explicit consented to these provisions will be enrolled in the clinical trial. The name of the subjects and other confidential information are subject to medical professional secrecy and the regulations of the applicable local data protection regulations. During the clinical trial, subjects will be identified by means of a unique individual identification code (pseudonym). The final trial report, public trial registers as well as scientific publications will solely contain anonymized statistical data.

### Quality assurance / monitoring

The study will be continuously monitored by the clinical research associates of the IZKS. Monitoring will be done by personal visits, telephone and mail contacts by a clinical monitor according to standard operating procedures. All participating centers will be visited by the monitor to ensure compliance with study protocol, good clinical practice and legal aspects.

### Safety

In this trial a DSMB will monitor and supervise the progress of the trial (including the safety data and the critical efficacy endpoints at intervals), review relevant information from other sources, ensure adherence to protocol, advise whether to continue, modify, or stop this trial. Furthermore it will provide the funding organization with information and advice. DSMB will meet annually and on special request. The trial management will organize these meetings and provide all necessary information for the work of the data monitoring board.

### Trial status

The trial is ongoing and in the recruiting phase at the time of manuscript.

## Discussion

The TME within a modern multimodal treatment options for rectal cancer resulted in a tremendous improvement of oncologic outcome and cancer-specific survival while dysfunction rates however remained still high. In consequence maintaining quality of life receives particular attention among colorectal surgeons aiming for best performance of intraoperative nerve-sparing. This is reinforced by the fact that the incidence of colorectal cancer diagnosed in young adults did significantly increase as demonstrated by a recent retrospective cohort study in 393241 patients [18]. Based on current trends it was stated that in 2030, the incidence rate for rectal cancer will increase by 124.2 %, respectively, for patients 20 to 34 years and by 46.0 %, respectively, for patients 35 to 49 years in the United States.

The prerequisite for intraoperative pelvic autonomic nerve preservation is the nerve identification. Only a few authors reported their rather higher or lower nerve identification rates while many others do not specifically provide such information but state its difficulty [3, 19, 20]. In addition to knowledge about aggravating circumstances and confounding factors, the recognition of key zones at risk of harm to the autonomic nerves is another important step for improvement of the nerve-sparing technique regardless of whether TME is performed minimally invasive or open via a transabdominal or transanal approach [21, 22]. In order to master intraoperative nerve-sparing the surgeon must rise to these challenges and take the lead in shifting towards aiming for a more sustainable quality of life.

The start however must be made in the informed consent discussion. A recent survey of rectal cancer patients undergoing surgery demonstrated that about 50 % of patients could not recall a preoperative discussion of risks to urinary, sexual and bowel function. Interestingly, they did desire more information regarding the occurrence of these possible dysfunctions than information on cure rate, need for second surgery, or the ability of surgery to treat their symptoms [23].

The pIONM may offer improvement of intraoperative nerve visualization and could be particularly beneficial with regard to all the confounding factors. Nevertheless, up to now there are no data from prospective randomized studies for comparing the functional outcome after TME for rectal cancer with and without pIONM. The aim of the present study is to evaluate whether pIONM is a valuable method for maintaining patient’s urogenital and anorectal function. This conducted prospective randomized multicenter trial will thereby demonstrate the efficacy, accuracy and safety of pIONM. Furthermore, potential advantages or disadvantages of this method can be analyzed. The study might also help to identify patients who would particularly benefit from pIONM.

With a view to primary prevention, pIONM may represent a useful tool for improvement of the nerve-sparing surgical technique in the minor pelvis. The additional quality assurance of pelvic autonomic nerve preservation after TME with predictability of postoperative urogenital and neurogenic ano(-neo)rectal dysfunctions could serve a secondary preventive function in enabling timely delivered commencement of causal urological-/sexological and proctological treatments with the potential for an improved prognosis in those patients with functional disturbances.

This is the first randomized multicenter trial comparing urogenital and anorectal functional outcome in rectal cancer patients undergoing TME with and without pIONM. If indeed found to be beneficial, pIONM will offer maintenance of patients’ quality of life and possibly decrease the amount of diagnostic and treatment costs of postoperative functional disturbances.

### Ethics approval and consent to participate

The trial is conducted according to ICH-GCP and the principles of the Declaration of Helsinki in its latest version. It was approved by the ethics committee of the State Chamber of Medicine Rhineland Palatinate, Germany under number 837.165.11 (7707) of the University Medicine of the Johannes Gutenberg University Mainz and subsequently by the other local ethics committees (Ethics Committee of Ludwig Maximilian University of Munich, 189-15; Ethics Committee of University of Lübeck, 14-231; Ethics Committee of Albert Ludwig University of Freiburg, 149/14; Ethics Committee of Friedrich-Alexander University Erlangen-Nürnberg, 156_15 Bc; Ethics Committee of University Medical Center Göttingen, 26/9/13; Ethics Committee of Hannover Medical School, 2131-2014; Ethics Committee of University Hospital of Leipzig, 347-15-05102015). Patients giving consent for participation sign the ethically approved patient informed consent.

### Consent for publication

Not applicable

## Abbreviations

CRF: case report form
CRM: circumferential resection margin
DSMB: Data Safety Monitoring Board
EMG: electromyography
FSFI: Female Sexual Function Index
IAS: internal anal sphincter
IIEF: International Index of Erectile Function
IPSS: International Prostate Symptom Score
ITT: intention-to-treat
IZKS: Interdisciplinary Center for Clinical Trials
LOCF: last observation carried forward
pIONM: pelvic intraoperative neuromonitoring
Qol: Quality of life index
TME: total mesorectal excision
WVS: Wexner-Vaizey score
